# Silk Fibroin-Based Biomaterials for Hemostatic Applications

**DOI:** 10.3390/biom12050660

**Published:** 2022-04-30

**Authors:** Md. Tipu Sultan, Heesun Hong, Ok Joo Lee, Olatunji Ajiteru, Young Jin Lee, Ji Seung Lee, Hanna Lee, Soon Hee Kim, Chan Hum Park

**Affiliations:** 1 Nano-Bio Regenerative Medical Institute (NBRM), Hallym University, Chuncheon 24252, Korea; tipubge@yahoo.com (M.T.S.); heesun181025@gmail.com (H.H.); vudckd@hanmail.net (O.J.L.); ajiteruolatunji@gmail.com (O.A.); skws789@naver.com (Y.J.L.); dbrghrl@naver.com (J.S.L.); dlgkssk1995@naver.com (H.L.); soonheekkim@gmail.com (S.H.K.); 2 Department of Otorhinolaryngology-Head and Neck Surgery, Chuncheon Sacred Heart Hospital, Chuncheon 24253, Korea

**Keywords:** silk fibroin, hemostatic agent, powder, sponge, sealant, medical application

## Abstract

Hemostasis plays an essential role in all surgical procedures. Uncontrolled hemorrhage is the primary cause of death during surgeries, and effective blood loss control can significantly reduce mortality. For modern surgeons to select the right agent at the right time, they must understand the mechanisms of action, the effectiveness, and the possible adverse effects of each agent. Over the past decade, various hemostatic agents have grown intensely. These agents vary from absorbable topical hemostats, including collagen, gelatins, microfibrillar, and regenerated oxidized cellulose, to biologically active topical hemostats such as thrombin, biological adhesives, and other combined agents. Commercially available products have since expanded to include topical hemostats, surgical sealants, and adhesives. Silk is a natural protein consisting of fibroin and sericin. Silk fibroin (SF), derived from silkworm *Bombyx mori*, is a fibrous protein that has been used mostly in fashion textiles and surgical sutures. Additionally, SF has been widely applied as a potential biomaterial in several biomedical and biotechnological fields. Furthermore, SF has been employed as a hemostatic agent in several studies. In this review, we summarize the several morphologic forms of SF and the latest technological advances on the use of SF-based hemostatic agents.

## 1. Introduction

Uncontrolled hemorrhage from military and civilian trauma accounts for the major cause of death during the prehospital era. However, effective control of blood loss at the injury site after trauma has been shown to significantly reduce mortality [1]. Hemostasis is the natural and physiological process that controls blood loss after trauma by clot formation and slowing blood flow to affected sites while maintaining normal blood flow elsewhere in the circulation thus, considered as the first stage of wound healing. Furthermore, Ingrid Hrachovinová defined hemostasis as a biological system wherein the balance between procoagulant and anticoagulant processes help maintain blood flow in intact blood vessels and, if needed, create thrombi in damaged vessels [2]. The hemostatic response must be quick, localized, and carefully regulated after disruption of a blood vessel wall. Abnormal bleeding or thrombosis (i.e., nonphysiologic blood clotting not required for hemostatic regulation) may occur when specific elements of these processes are dysfunctional or missing.

The cascade of events that occur immediately after injury to the blood vessels and preceding wound healing can be grouped into three major components: vasoconstriction, primary hemostasis, and secondary hemostasis [3]. These events occur simultaneously to stop blood loss from the injured vessel. Natural vasoconstrictor hormones such as epinephrine (a general vasoconstrictor hormone) and thromboxane (a localize vasoconstrictor hormone) are immediately released when a blood vessel ruptures. The vasoconstrictor hormones cause the blood vessel muscular walls to contract, thus narrowing the blood vessel lumen, reducing the blood flow, and subsequently blood loss.

**Figure 1:**
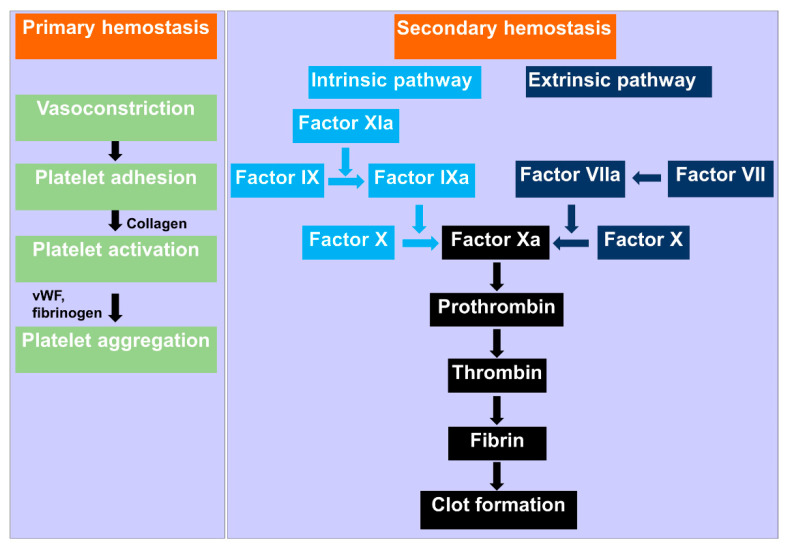
Schematic mechanism of hemostasis. Primary hemostasis starts with vasoconstriction at the injury site, and platelet aggregates due to the interaction of fibrinogen and Willebrand factor (vWF). Secondary hemostasis involves the formation of insoluble, cross-linked fibrin via stimulated coagulation factors, especially thrombin.

Primary hemostasis refers to platelet aggregation and its subsequent plug formation (Figure 1). Platelets are activated in a multifaceted process when exposed to the subendothelial matrix. As a result, they adhere to each other (aggregation) and the injury site, thereby plugging the injury. Specifically, when blood flows into the subendothelial of the vessels wall containing collagen due to injury, the constitutively active platelet collagen receptor, glycoprotein VI (GPVI) becomes activated by collagen (ligand). Activation of GPVI is critical to platelets adhesion to the subendothelial matrix of the blood vessel at the injury site [4]. Furthermore, the high shear stress exerted by blood flow (during vasoconstrictions after injury) on the von Willebrand factor (VMF) increases its affinity for GPIbα domain of GPIb-IX-V receptor [5]. This interaction initiates platelets aggregation. The feedback mechanism of aggregated platelets causes further aggregation of nearby platelets and propagation of platelet plug [6].

Secondary hemostasis is a complex process that involves the formation of insoluble, cross-linked fibrin via stimulated coagulation factors, especially thrombin [3] (Figure 1). In this hemostasis process, the cascade of coagulation serine proteases is completed in the cleavage of soluble fibrinogen by thrombin. The resultant insoluble fibrin from the thrombin cleavage form constructs a cross-linked fibrin mesh at the injury site. Fibrin formation occurs concurrently with platelet aggregation [7,8]. This cascade is not initiated in the healthy and intact blood vessels. Secondary hemostasis includes the extrinsic pathway diverging the intrinsic pathway to form the common pathway and formation of the fibrin. Blood is exposed to extravascular tissue during the injury of the vascular system. The extravascular tissue is rich in a cofactor, tissue factor (TF), of the serine protease factor VIIa [9]. In the extrinsic pathway of the coagulation, the complex of TF and factor VIIa initiates the factor X and IX. In the presence of the cofactor VIIIa, factor IXa activates the factor X. Factor Xa then activates prothrombin in the presence of its cofactor factor Va to generate thrombin [10]. Thrombin, an essential serine protease in the coagulation cascade, performs various critical reactions, including cleaves of fibrinogen to generate insoluble fibrin [11]. Platelets are activated by thrombin via the cleavage of PAR1 and PAR4 [12]. In the intrinsic pathway, thrombin initiates factor XI, which triggers factor IX. In this pathway, thrombin also activates cofactors VII and V [11]. Fibrin alleviates the primary platelet plug, mainly in larger blood vessels, wherever the platelet plug is inadequate alone to stop bleeding. Thrombosis or pathological hemorrhage can arise whenever the hemostatic progression is dis-regulated [3]. The frequent occurrence of anomalies associated with these physiologically regulated processes has given rise to the need for external hemostatic agents.

Externally applied hemostatic agents can be administered to simple wounds, puncture and exposed wounds, bruises, surgical lacerations, and blunt force trauma [13]. There are several hemostatic agents available in the present day, such as those composed of cotton, oxidized cellulose, oxidized regenerated cellulose, collagen, gelatin, alginate, chitosan, polylysine, polyarginine, and poly (propylene oxide) (PPO) [14,15]. To exhibit enhanced hemostasis, hemostatic agents should display biocompatibility and the ability to reduce blood loss, provide a favorable hemostatic condition for long-term, confer protection against pathogen and dust, maintain structure for gaseous permeation, the biodegradability of the material to escape disrupting the lesion site, easy application, and removal by non-specialized personnel [16,17,18,19]. A number of hemostatic materials have been developed in the form of spray, powder, gels, foam, sponge, tourniquets, and tamponades for externally accessible and visible injuries [14]

SF is a natural polymer derived from silkworm *Bombyx mori*, consisting of 26 kDa light and 390 kDa heavy chain linked by a disulfide [20,21]. The amino acid sequences of SF include repetitive Gly–Ala–Gly–Ala–Gly–Ser repeats that can be formed a β-sheet structure by self-assembly [22,23]. SF has been applied as a functional biomaterial with excellent biocompatibility, robust mechanical properties, minimal immune reactions, controlled biodegradation and water permeability. For these reasons, SF has been fabricated into various forms for several applications including, films, sponges, electrospun mats, nano- or microparticles, and hydrogels [24,25]. Because of these backgrounds, SFs are being progressively studied for biomedical practices, such as skin, bone, ligament, cartilage, tendon, dental, cornea, tympanic membrane, nerve, artificial kidney, and bladder. SF has displayed degradability and non-toxicity with effective biosafety and can enhance cell adhesion, proliferation, differentiation, and migration of several cell types such as epithelial, endothelial, fibroblast, keratinocyte [24,26,27]. Due to these properties, SF is frequently applied to promote wound dressings [28], surgical sutures, and other aspects of wound treatments [29]. The addition of SF to other hemostatic materials can advance coagulation activity reduce bleeding time and bleeding volume [30]. Therefore, in the recent past, several studies conducted on SF for the hemostatic application in several forms including, powder, sponge, and sealant. This review focuses on several morphologic forms of SF and the cutting-edge technological advances of SF-based hemostatic agents used in hemostasis.

## 2. SF in Different Forms for Hemostatic Applications

SF can be converted to a wide range of forms such as solutions, powder, fibers, films, sponges, hydrogels, and sealants through the various treatment process. SF-based biomaterials have been extensively applied in various forms in different hemostatic applications.

### 2.1. SF Powder

The hemostatic powder is an inorganic particle that does not adhere to non-bleeding surfaces, affecting only active bleeding areas [31,32]. The powder exhibits adhesive properties and dehydrates the tissue by absorbing water molecules, causing its volume to increase [15,32]. It acts as a physical barrier when in contact with water and concentrates clotting factors at the bleeding site, which then forms a clot [33]. Although they are not a magic powder that can be casually applied somewhere near the wound and left to work, they have a wide range of clinical applications [34,35], including inaccessible and irregular injuries [36]. In the recent past, numerous hemostatic agents such as HemCon [37,38] chitosan dressing and QuickClot zeolite powder [39,40] have been developed to stop bleeding and fatality before migration to decisive care. These hemostatic agents are the most common hemostatic powders based on porous structure. The porous form of each powder improves water absorption and local thrombin concentrations. It has been reported that the zeolite-based QuickClot material exhibited improved hemostatic characteristics in some big animal trials and field conditions. In a previous study, it has been shown that the QuickClot attained 100% survivability in a lethal groin injury model of swine, but the efficacy was adversely affected due to the increased amount of residual moisture [39]. It also has an extreme exothermic effect with the highest temperature of 100 °C that can cause secondary tissue injury and foreign-body immune reaction [41,42]. HemCon, a chitosan-based dressing, has been shown excellent biocompatibility with good biodegradability and can increase the wound-healing process. A previous study showed that a chitosan dressing significantly enhanced hemostasis over gauze, as demonstrated by better survival in the swine model of venous hemorrhage and hepatic injury [37]. However, HemCon chitosan dressing as pads are challenging to conform to deep, narrow, or wounds of irregular shapes [43]. Therefore, the challenge still remains to develop an effective hemostatic material that can control the bleeding with minimal damage.

In several recent studies, SF powder has been shown to have excellent homeostatic properties both in in vitro and in vivo [44]. Lei et al. recently showed superior hemostatic efficacy of the low molecular weight SF (LMSF) in vitro and in vivo [30]. LMSFs were prepared via hydrolysis of SF in a ternary solvent system of CaCl_2_/H_2_O/EtOH in their study. LMSF solutions were dried at 150 °C for 24 h to make LMSF powder. The absorption efficacy and Platelet adhesion were determined using the whole blood of New Zealand Rats. LMSF showed significantly higher absorption capacity than the gauze, and this higher absorption capacity might be attributed to the gelation phenomenon. In the case of Platelet adhesion test, a significant number of platelets adhered on LMSF than gauze at the same experimental time. They also evaluated the hemostatic efficacy of the LMSF in a severe liver injury model of rats and found a rapid blood clotting activity with decreased blood loss and bleeding time. They did not mention the exact hemostatic mechanism of the LMSF. They speculated that the LMSF had the preference to concentrate the cellular and plasma components in the blood at the site of the wound.

Hemostatic powder made of microspheres such as chitosan offers some antimicrobial properties. Still, it needs to be dissolved in a strong acid solvent due to its poor solubility, making its limited clinical applications [45]. In some cases, it also induces a similar allergic reaction associated with seafood. It has been known that the addition of SF to other hemostatic materials can enhance coagulation activity, reduce bleeding volume and shorten bleeding time [28,30]. Huang et al. has been successfully developed a hemostatic microsphere with surface roughness of SF/sodium alginate (SA) microspheres for rapid hemostasis [44]. They fabricated this SF/SA hemostatic microsphere by emulsion cross-linking of SA and SF. Unlike standard porous microspheres, the surface morphology of SF/SA microspheres was rough, which enhanced the adhesion of surface erythrocytes and platelets, which helped prevent blood cells from entering the inside of the microspheres. The water absorption of SF/SA microspheres was more potent than that of SA, SF microspheres. This indicated that SF could improve the water absorption capacity of SA under some defined conditions [46]. It has also been demonstrated that these microshres have the most significant number of aggregated red blood cells and the fastest coagulation rates with the most substantial coagulation strength. SF/SA microspheres exhibited reduced bleeding volume and time with improved hemostatic efficiency in vitro and in vivo experiments. Therefore, SA and SF have a synergistic hemostatic effect when used in appropriate proportions. The rough surface microspheres that can strongly accelerate blood coagulation suggested that SF/SA microsphere can be a potential new hemostatic agent with excellent biosafety.

**Figure 2:**
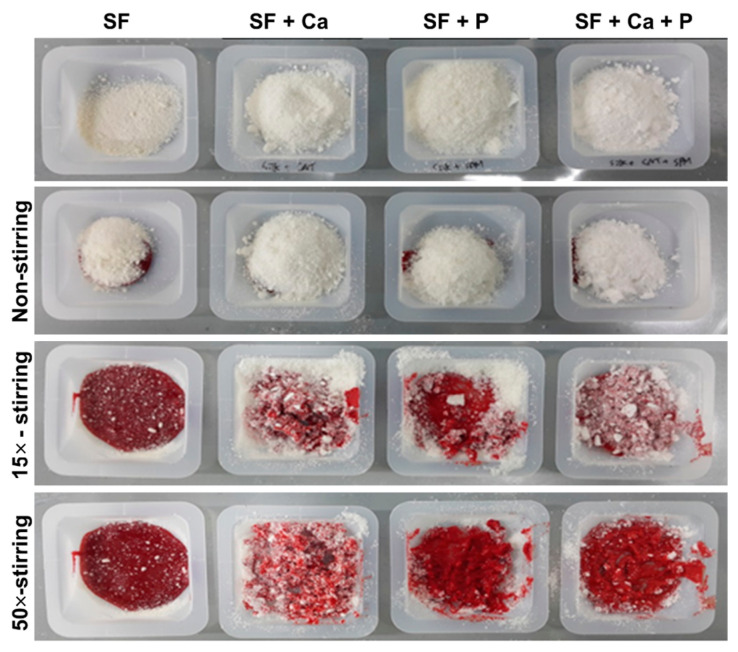
Evaluation of the in vitro blood coagulation effect of the SF-based powder. SF powders containing calcium (SF + Ca) and phosphoric acid (SF + P) exhibited improved blood coagulation when it reacted with them. Unpublished data.

Our laboratory successfully developed SF-based powders with micro-size particles containing calcium (Ca) and phosphoric acid (P). Our in vitro coagulation experiments demonstrated that the hemostatic effect is insignificant when SF has applied alone (Figure 2). SF powders containing calcium (SF + Ca) and phosphoric acid (SF + P) absorbed more blood and aggregates when it reacted with them. Silk-based powders containing calcium and phosphoric acid (SF + Ca + P) also showed enhanced blood clotting. Therefore, it can be attributed to the improved hemostatic effect achieved by the SF-based powder due to the synergistic effect of SF, Ca, and P.

**Figure 3:**
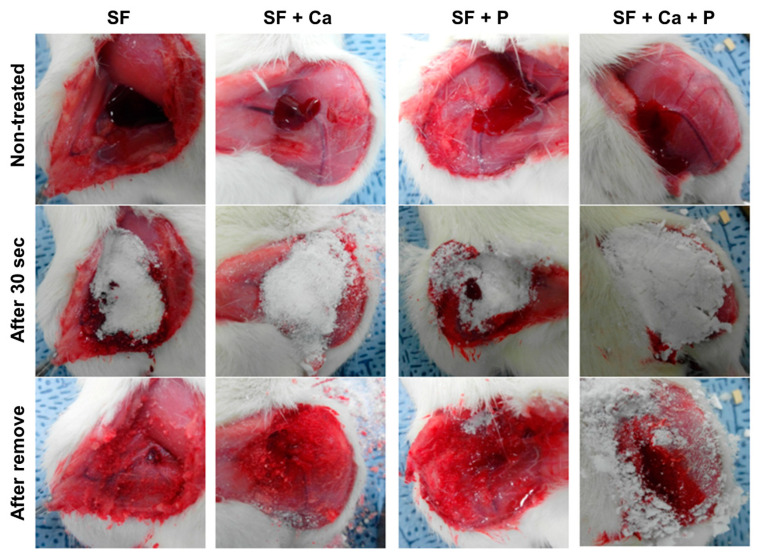
In vivo hemostasis of the SF-based powder using the femoral vein incision of the rat. After the femoral vein incision, SF-based powders were applied to stop bleeding for 30 s. Unpublished data.

We have examined the hemostatic effect of the SF-based powders in the rat femoral artery hemorrhage experiment. Bleeding in the femoral vein of Sprague Dawley rats was induced, and SF-based powders were treated. After 30 s, the bleeding was stopped in SF powders. SF + Ca and SF + P powders maintained bleeding after 30 s of treatment, but the hemostatic effect was observed after 1 min (Figure 3). Hemostasis containing both calcium and phosphoric acid (SF + Ca + P) continued bleeding after 30 s of treatment, but it was confirmed that hemostasis was completed after 1 min. This result suggests that silk fibroin-based powders are potential hemostatic agents.

### 2.2. SF Sponge/Film

Sponge scaffolds offer a precise network of interconnected pores with a high volume of surface area within a well-defined three-dimensional structure. Sponges have been extensively used for various biomedical applications, including tissue engineering [47] and drug delivery [48] due to their excellent properties such as porous structure, ease in fabrication, ability to absorb massive amounts of biological fluids or water, capability to act as natural tissue physical properties, and remarkable biocompatibility. Recently, sponges of natural or synthetic biomaterials such as polyvinyl alcohol, gelatin, and chitosan have emerged as attractive hemostatic materials due to their soft physical structure, high absorption capacity, large-scale production, and easily applicable to the bleeding site [49,50,51,52]. Among them, the chitosan-based sponge materials have been widely investigated for hemostatic applications due to their unique features, including no genetic toxicity, no cytotoxicity, no hemolysis, excellent biological compatibility, and biodegradable properties [53,54]. For example, HemCon (an American company) developed a hemostatic dressing named the HemCon Bandage, whose main ingredient was chitosan.

Porous sponges are essential to tissue engineering materials, and regenerated SF also has been exploited in the fabrication of porous sponges [55,56,57,58,59,60]. In the recent past, SF as a hybrid sponge in combination with other polymers has been fabricated due to the fragile nature of the SF sponge and applied successfully in several studies for tissue engineering and hemostatic purposes [61,62]. Several studies have shown that an SF sponge can make rapid blood coagulation when mixed with blood [63,64]. SF porous sponges can be fabricated by gas-forming, porogens, freeze-drying, and electrospun technology [65,66,67,68]. Wei et al., developed a gelllable sponge combined with SF and polyethylene glycol (PEG) for hemostasis using freeze-drying process [69]. They demonstrated both the haemostatic properties and hemostasis mechanism of SF-PEG sponge in their study. The hemostatic effects of the SF-PEG sponge were better than that of clinically used gelatine sponges using liver wound models of rabbits. SF-PEG sponge physically blocked the bleeding port upon SF gel formation in the presence of PEG. SF might also affect the blood coagulation process because it was demonstrated that SF could significantly activate platelets in vitro, enhance platelet aggregation and adhesion, and promote blood coagulation.

**Figure 4:**
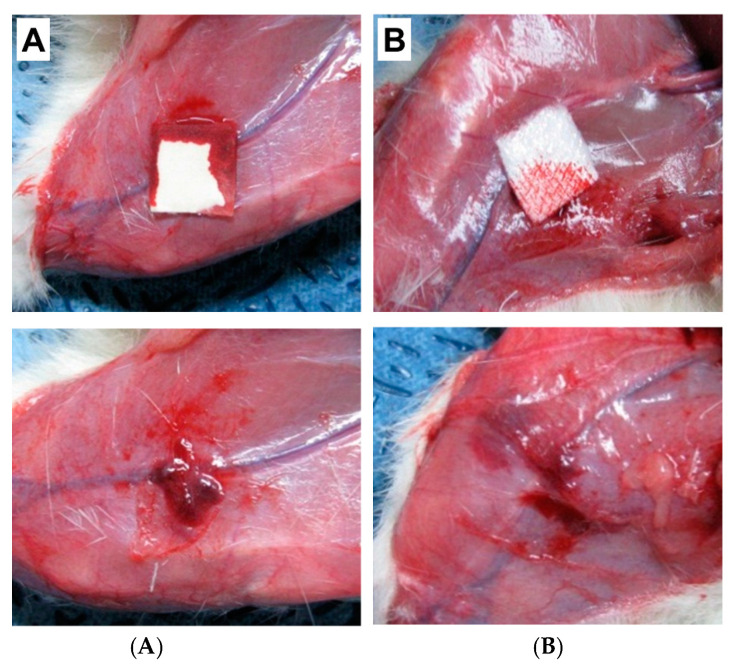
Evaluation of the hemostasis of the SF/Gel/PVA sponges using rat femoral hemorrhage injury model. Before removing hemostatic agents (upper panel) and after (lower panel). (**A**) ChitoClot^®^, (**B**) SF/Gel/PVA [70].

**Figure 5:**
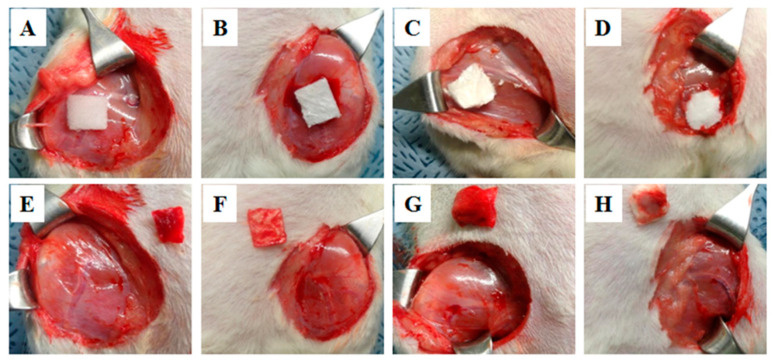
Hemostatic effect of SF-based sponges in a femoral artery wound of rat model. (**A**,**E**) Avitene, (**B**,**F**) Duck’s feet collagen (DFC), (**C**,**G**) Silk and (**D**,**H**) DFC/Silk [62].

In our previous study, we have developed a hemostatic agent in the combination of three biomaterials; SF, Gelatin (Gel), and Polyvinyl alcohol (PVA) [70]. We have evaluated the hemostatic efficacy of the SF/Gel/PVA sponge against the commercially available hemostatic pad (ChitoClot^®^). We found that SF/Gel/PVA sponge exhibited better hemostatic properties than ChitoClot^®^ when tested in an injured model of rats (Figure 4). Recently Seo et al. have been developed an SF-based sponge with duck’s feet collagen for hemostatic application [62]. The hemostatic properties of the sponges were measured by both in vitro (whole blood clotting) and in vivo test (rat femoral artery hemorrhage experiment). The inclusion of SF to duck’s feet collagen sponge showed improved blood clotting efficacy than commercially available hemostatic agent Avitene in vitro. However, a similar hemostatic property was observed between SF-based sponges and Avitene during rat femoral artery hemorrhage experiment (Figure 5). Although they observed a similar hemostatic property between SF-based sponges and Avitene, they suggested that SF-based sponges could be effectively applied for hemostatic applications. SF sponge provides several advantages over other hemostatic materials, including easily obtainable, high swelling ratio, sterilizable, well-interconnected pores with a great extent of surface area within a well-defined three-dimensional volume, which allows it to absorb a large amount of blood or fluid during the hemostatic process. Therefore, it could be clinically applicable for hemostasis.

SF films are a candid biomaterial of choice for biomedical applications owing to their intrinsically less complex character regarding scaffold development. SF film can be easily fabricated using casting, spin-coating, and layer-by-layer deposition techniques [71,72,73]. SF films have been vastly applied for wound dressing materials in numerous studies. Recently, Huang et al. reported a light-triggered SF-based film with effective antibacterial activity for hemostatic applications [74]. In this study, they developed a light-responsive SF film to effectively manage liver hemorrhage with effective treatment against S. aureus on near-infrared (NIR) irradiation. After UV exposure, the SF film achieved excellent tissue adhesion and hemostasis within 2 min in vivo. Therefore, this film could be used as a hemostatic agent for rapid hemostasis with effective antibacterial activity to reduce blood loss and improve clinic survival.

### 2.3. SF Sealant

In the recent past, tissue sealants, to stop bleeding, have become progressively important regards to the expansion of surgery with minimal invasion. Therefore, medical glues such as sealants, adhesives, and hemostatic agents have become more crucial in surgical practice [75]. Among the medical glues, surgical sealants have gained significant attention as promising replacements for ease of operability and improved sealing with negligible traumatic closure [76,77]. The ideal medical sealants should be easy to use, safe, and enhanced efficacy with low cost and fulfill the regulatory requirements. However, clinically available tissue sealants, including polyurethane-based tissue adhesives, polyethylene glycol-based tissue adhesives, cyanoacrylate synthetic glues, and fibrin sealants, are far from ideal owing to their toxicity poor adhesion under wet conditions such as bleeding tissues [76,78,79,80]. For instance, the clinical translation of the cyanoacrylate limit due to the release of its degraded toxic products after solidification upon exposure to blood and more rigid than native tissues [79,80]. On the other hand, the fibrin sealant cannot tolerate the forces in dynamic conditions (e.g., shear of blood flow, beating heart) because of their poor adhesion strength to bleeding tissues [76]

SF-based medical glues have been reported as an ideal hemostatic sealant in several studies, for instance, SF combined with catechol-functionalized SF containing PEG and chemically active polyethylene glycol (PEG) [81,82]. Serban et al. developed a colloidal type of glue in combination with SF and chemically active polyethylene glycol (PEG) by a chemical reaction between thiol and maleimide [82]. They explored that SF-PEG-based materials could be an excellent hemostatic sealant due to their biological, physical, and mechanical properties. Catechol-functionalized SF-PEG-based adhesives have also been developed for the application as biodegradable adhesives and sealants [81].

**Figure 6:**
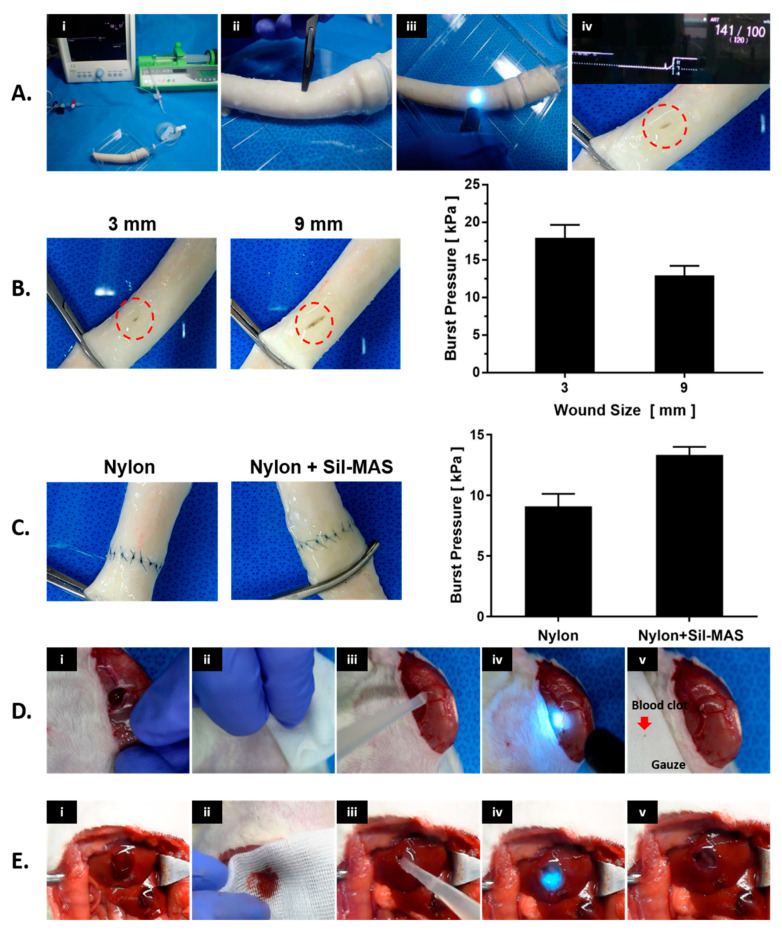
Vascular adhesive and hemostatic effect of Sil-MAS on wet surfaces. (**A**) Images of ex vivo burst pressure test process for Sil-MAS. (**i**) The setup for the burst pressure experiment, (**ii**) Opening in the middle of the aorta, (**iii**) Appication of Sil-MAS on the wound site and UV exposure. (**iv**) Completion of the aorta burst pressure test. (**B**) Porcine aorta burst pressures on the incision shielded with Sil-MAS. (**C**) Porcine aorta burst pressures after end-to-end anastomosis by Nylon suture alone and Nylon suture with Sil-MAS. (**D**) In vivo vascular closure experiment of Sil-MAS. (**i**) Femoral artery incision and bleeding, (**ii**) Slight hemostasis using gauze, (**iii**) Use of Sil-MAS on the incision site and UV exposure, (**iv**) Cessation of bleeding, (**v**) Confirmation of hemostasis. (**E**) Images of the sealants used in an in vivo liver parenchymal injury model. (**i**) The bleeding from the injury site rose after a circular tissue extraction with a 0.5 cm long and 0.5 cm deep wound on the rat liver surface. (**ii**) After wiping the wound using gauze for slight hemostat, (**iii**) Sil-MAS (or Fibrin glue) was employed to the resected site, (**iv**) cross-linked by UV (for Sil-MAS) and thrombin (for Fibrin glue), and (**v**) bleeding stopped. [84].

**Figure 7:**
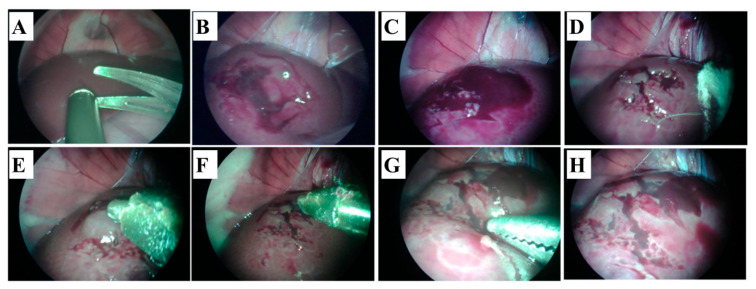
Hemostatic effect of Sil-MAS on the rabbit liver laceration model. (**A**,**B**). Creating of the liver laceration (deep and superficial) by endoscopic scissors and suction. (**C**) Bleeding and hematom on the surface of the liver. (**D**) After briefly pressing with gauze on the bleeding liver laceration wounds (**E**) Application of the Sil-MAS (1.0 cc) using self-made laparoscopic Sil-MAS device. (**F**) UV (20 s) exposure via fiber optic lens in the device. (**G**) Examination of the re-bleeding or outflow of blood at the lesions using endoscopic forceps. (**H**) Completion of the adhesion and gelation of Si-MAS on liver laceration lesion [84].

Poor adhesion of the hemostatic sealant to wet tissue surfaces, particularly in extremely dynamic biological conditions, is vital in clinical arenas but remains highly challenging. To address this problem, an SF-based sealant by introducing tannic acid into SF (SFT) has been developed by Bai et al. for instant hemostasis of bleeding tissues [83]. They showed rapid sealing of severely bleeding tissues under wet and extremely dynamic environments using in vivo experiments in rat models. Although this method has a long gelation time, the engineered SFT also demonstrated excellent biocompatibility and biodegradability with effective antibacterial protection. Recently, our group has fabricated a methacrylated SF sealant (Sil-MAS) sealant that can be applied as a hemostatic agent or adhesive [84]. Sil-MAS showed excellent physical properties through in vitro mechanical tests and ex vivo aorta pressure tests (Figure 6). Sil-MAS exhibited an excellent hemostatic and adhesive capability, with high biocompatibility via in vivo biological tests on rats’ skin, liver, and blood vessels. Sil-MAS also intensely contributed to quicker wound healing capacity than commercially available materials. In addition, we demonstrated a successful proof of concept that Sil-MAS could use as an ideal photocuring laparoscopic medical glue in a laceration liver and stomach serosa model of rabbit by a homemade endoscopic device (Figure 7). Therefore, we suggested that Sil-MAS could be used as a suitable sealant for versatile medical application to supplement of existing hemostatic sealants, adhesive or glue.

## 3. Conclusions

Control of the bleeding in the surgical room remains an issue despite a vast advancement in the recent past. FDA has approved several hemostatic agents those are satisfied the practical requirements for medical practices. Still, there is a need to develop an improved product regarding material properties. Several synthetic and natural materials in various forms such as powder, sponge, adhesive, and sealant have been applied as an alternative hemostatic agent, including zeolite, PVA, PEG, HA and chitosan. SF (FDA approved) is one of the standard natural biomaterials applied as a luxurious textile material for over 4000 years. “The biocompatibility of the SF protein has been comprehensively explored [85,86,87], and there is no bioburden including allergic reaction associated with SF when compared with other biopolymers such as collagen and chitosan. SF also exhibits relatively slow degradation due to its β-sheet structure both in vitro and in vivo compared with collagens and several other biopolymers [85,88]. SF in various formats, including powder, sponge, films, electrospun fibers, and hydrogels has been comprehensively employed for a wide range of applications, such as drug wound healing, delivery, and tissue engineering due to their excellent biocompatibility, less inflammatory response to host tissue, slow biodegradation rates, cost-effective and ease of use. Therefore, it has been discovered to be a promising standard biomaterial in various forms, such as powder, sponges, films, adhesive/sealant in various hemostatic applications.

Although SF-based biomaterials have been extensively applied for hemostasis, there are very few studies that explored the actual mechanism of SF in hemostasis. Several studies have been conducted using SF as solutions, scaffolds, and films for hemostatic application to evaluate the mechanism of SF in hemostasis [63,64]. These studies only focused on one or a few parameters of the blood coagulation cascade, including activation of the partial thromboplastin time, prothrombin time, and platelets activation due to the interaction between blood and biomaterial [89,90,91]. An early study demonstrated the hemostasis achieved by SF biomaterial through the platelet-mediated activation of the blood coagulation cascade [92]. Several studies have been shown that an SF sponge can enhance blood coagulation when it mixed with blood [63,64]. However, most existing hemostatic agents that contain SF proteins are blended with other hemostatic agents such as chitosan, collagen, and gelatin. Therefore, it is difficult to elucidate the role of SF in hemostasis. Wei et al. recently developed a gellable SF-polyethylene sponge for hemostasis [69]. Their study exhibited that SF promoted blood coagulation through significantly platelets activation, platelet aggregation, and adhesion in vitro. However, the study of SF-based hemostatic agents is still at an early stage; more inclusive studies are needed to elucidate the actual mechanism of SF in hemostasis in the future.

“SF is a well-established textile fiber, and about 1000 metric tons of silk are produced and processed yearly [93]. A simple alkali or enzyme-based degumming process is regularly used to produce sericin-free SF-based biomaterials. SF-based biomaterials for hemostatic application are also economically advantageous and sustainable due to the available infrastructure of traditional silk textile industries for the large-scale processing of SF. However, SF has drawbacks such as brittleness, easy fragmentation, and difficulty in generating a uniform thickness. Further studies are warranted to create a new array of SF-based hemostatic agents and improve the potential effect of SF for different types of hemostatic applications.

## Data Availability

The authors declare that all data of this study are available within the article from the corresponding author on reasonable request.
